# A 10 year comparative study of caesarean deliveries using the Robson 10 group classification system in a university hospital in Austria

**DOI:** 10.1371/journal.pone.0240475

**Published:** 2020-10-16

**Authors:** Taja Bracic, Isabella Pfniß, Nadja Taumberger, Kaltrina Kutllovci-Hasani, Daniela Ulrich, Wolfgang Schöll, Philipp Reif

**Affiliations:** Department of Obstetrics and Gynecology, Medical University of Graz, Graz, Austria; Azienda Ospedaliero Universitaria Ospedali Riuniti di Ancona Umberto I G M Lancisi G Salesi, ITALY

## Abstract

**Objective:**

The Robson ten group classification system is used as a global standard for assessing, monitoring and comparing caesarean delivery (CD) rates within and between maternity services. Our objective was to compare the changes of CD rates at our institution between the years 2008–2010 and 2017–2019 using the Robson ten group classification system.

**Study design:**

Data was collected retrospectively and all women were classified using the obstetric concepts and parameters described in the Robson ten group classification system.

**Results:**

During 2008–2010 7,832 deliveries were performed, increasing to 9,490 in 2017–2019. The CD rate also increased from 29.1% to 32.2% (p<.05) during this 10 year period.

In both observed periods group 5 (single cephalic multiparous women at term with a previous CD) was the largest contributor to the overall CD rate accounting for 20.2% of all CD during 2008–2010 and increasing to 26.9% in 2017–2019 (p<.001). The overall size of group 5 also increased from 8.3% to 11.6% (p<.001). Furthermore, an increase in CD rate in group 7 (multiparous women with a single breech pregnancy, including women with a uterine scar) from 92.9% to 98.2% (p = .752) could be observed. In group 8 (women with multiple pregnancies, including women with a uterine scar) a slight shift towards vaginal delivery (VD) can be reported with CD rates decreasing from 82% to 79.2% (p = .784). There was no observed difference with CD rates in group 1 although the group size decreased from 29.4% in 2008–2010 to 24.2% in 2017–2019 (p<.001). The CD rate in group 10 experienced a slight elevation, in 2008–2010 46.2% were delivered per CD and in 2017–2019 48.8% (p = .553). The overall size of group 10 decreased, contributing 8.9% in 2008–2010 and 8% in 2017–2019 (p<.05) to the overall birthrate.

**Conclusion:**

The biggest contributors to the CD rate in our hospital remain multiparous women at term with a previous CD. The CD rates, as well as the overall size of this group, keep rising, resulting in a need to establish more effective ways to motivate women with one previous CD towards vaginal birth after caesarean delivery (VBAC). Furthermore, the CD rate in preterm deliveries is increasing and approaching 50%. This illustrates the need to discuss whether CD is the appropriate mode of delivery in half of the preterm infants.

## Introduction

Caesarean delivery is one of the most common surgical procedures in medicine. Initially developed as an emergency, lifesaving procedure its use has expanded dramatically in the last decades all over the world. Optimized technique and low morbidity and mortality rates resulted in a rising number of indications [1].

The reported continuously increasing rates of caesarean delivery (CD) have multifactorial reasons and are associated with changes in maternal characteristics and professional practice styles, increasing malpractice pressure, as well as social and cultural factors [2,3].

Due to worldwide increasing CD rates obstetricians are being faced with a number of short- and long-term complications which especially become relevant in multiparous women with 1 or more CD [4].

Differences between low- and high-income countries are still present, resulting in low-income countries demonstrating significantly lower CD rates than the world average.

A recent analysis by Betran et al [2] established a worldwide CD rate of 18,6% in 150 countries. In contrast in Europe 25% of all deliveries are being done per caesarean delivery [2].

Since the benefit of reducing maternal, neonatal and infant mortality at CD rates higher than 10–15% is yet to be proven, the indications should be considered carefully. In 2015 WHO has recommended the Robson classification as an internationally applicable caesarean delivery classification system for hospitals to regularly control the effect of their actions taken to optimize the use of caesarean delivery as well as to identify, analyse and focus interventions on specific groups [5].

The ten group classification system introduced by M. Robson in 2001 classifies women into 10 groups based on 5 characteristics: number of foetuses, foetal presentation, parity, onset of labour, and gestational age [6].

The aim of this study was to analyse changes of caesarean delivery rates at our department for obstetrics during the years 2008–2010 and 2017–2019 and to categorize them into the ten group classification system. The results should help us to determine which patients are the leading contributors to rapidly rising CD rates, as well as motivate us to develop strategies how to prevent a further increase in the future.

## Material and methods

This retrospective study analyses a data set of 7832 births that took place between 1^st^ January 2008 to 31^st^ of December 2010, as well as 9490 births recorded from 1^st^ January 2017 to 21^st^ November 2019 at the department of obstetrics of Medical University of Graz (MUG), Austria. The study was reviewed and approved by the ethics committee of the Medical University of Graz with the IRB number 00002556 before the study began. The committee waived the requirement for informed consent. With an average of 3500 deliveries per year the obstetrical unit of the MUG is one of the biggest in Austria. Upon arrival at the ward the patient’s data is automatically recorded and documented in a digital patient record system (View point, Version 5.6., GE Healthcare, Solingen, Germany).

The View point database is a computer based system widely used in obstetric departments in Austria for antepartum, intrapartum and postpartum documentation. A wide range of motherly and foetal characteristics can be documented, as well as the labour process and the postpartum period, including ultrasound images. On demand the characteristics of interest (in our case: number of foetuses, foetal presentation, parity, onset of labour, gestational age) recorded in a specific period of time, can be extracted from the record system. Before analysis data was automatically fully anonymized. Each birth was assigned a number to assure anonymization. All births that were missing one or more of the 5 needed characteristics were excluded from the study. The births were categorized in 10 groups, additionally the groups 2,4 and 5 were subcategorized in 2 subgroups as proposed by Robson (Table 1).

**Table 1:** The ten group classification system.

1	Nulliparous women with a single vertex pregnancy, at ≥37 weeks gestation in spontaneous labour
2	Nulliparous women with a single vertex pregnancy, at ≥37 weeks gestation, who had labour induced or who had CD before labour
2a	Nulliparous women with a single vertex pregnancy, at ≥37 weeks gestation, who had labour induced or who had CD before labour
2b	Nulliparous women with a single vertex pregnancy, at ≥37 weeks gestation, who had CD before labour
3	Multiparous women, without a uterine scar, with a single vertex pregnancy at ≥37 weeks gestation in spontaneous labour
4	Multiparous women, without a uterine scar, with a single vertex pregnancy at ≥37 weeks gestation, who had labour induced or who had CD before labour
4a	Multiparous women, without a uterine scar, with a single vertex pregnancy at ≥37 weeks gestation, who had labour induced
4b	Multiparous women, without a uterine scar, with a single vertex pregnancy at ≥37 weeks gestation, who had CD before labour
5	Multiparous women, with at least one previous uterine scar with a single vertex pregnancy at ≥37 weeks gestation
5a	Multiparous women, with one previous uterine scar with a single vertex pregnancy at ≥37 weeks gestation
5b	Multiparous women, with more than one previous uterine scar with a single vertex pregnancy at ≥37 weeks gestation
6	All nulliparous women with a single breech pregnancy
7	All multiparous women with a single breech pregnancy, including women with a uterine scar
8	All women with multiple pregnancies, including women with a uterine scar
9	All women with a single pregnancy with a transverse or otherwise abnormal presentation, including women with a uterine scar
10	All women with a single vertex pregnancy at < 37 weeks gestation, including women with a uterine scar

Introduced by M.Robson in 2001 and recommended by the WHO in 2015 for analysing caesarean delivery rates.

The CD rate was calculated by dividing the number of CD in each group over the total number of births in this particular group. Group size was determined as the number of births in one group divided with all births in the observed time period. Results are shown as percentage.

The statistical analysis was performed with IBM SPSS Statistics for Windows, Version 26 (IBM Corp., Armonk, N. Y., USA). For the comparison of the two time periods the Chi Square Test was performed. P-Values <.05 were labelled as significant.

## Results

In 2008–2010, 7832 of 7992 births were eligible for analysis, during 2017–2019 9490 of 10035 met the criteria. In the first period in 2% and in the second period in 5.4% data was incomplete and therefore excluded. (Tables 2 and 3).

**Table 2:** Birth data 2008–2010.

	total births n = 7832	total CD n = 2280 (29,11%)	Size of group	CD rate in group	Absolute group contribution to overall CD rate	Relative Contribution of the group to overall CD rate
**1**	2302	378	29.39%	16.42%	4.83%	16.58%
**2**	1001	382	12.78%	38.16%	4.88%	16.75%
**2a**	915	296	11.68%	32.35%	3.78%	12.98%
**2b**	86	86	1.10%	100%	1.10%	3.77%
**3**	1922	87	24.54%	4.53%	1.11%	3.82%
**4**	676	119	8.63%	17.60%	1.52%	5.22%
**4a**	613	56	7.83%	9.14%	0.72%	2.46%
**4b**	63	63	0.80%	100%	0.80%	2.76%
**5**	649	460	8.29%	70.88%	5.87%	20.18%
**5.1**	588	399	7.51%	67.86%	5.09%	17.50%
**5.2**	61	61	0.78%	100%	0.78%	2.68%
**6**	209	208	2.67%	99.52%	2.66%	9.12%
**7**	112	104	1.43%	92.86%	1.33%	4.56%
**8**	245	201	3.13%	82.04%	2.57%	8.82%
**9**	19	19	0.24%	100%	0.24%	0.83%
**10**	697	322	8.90%	46.20%	4.11%	14.12%

Collected data of all births and caesarean deliveries performed at the department of obstetrics at Medical University Graz in the years 2008–2010 analysed using the ten group classification system by M. Robson.

**Table 3:** Birth data 2017–2019.

	total births n = 9490	total CD n = 3051 (32,15%)	Size of group	CD rate in group	Absolute group contribution to overall CD rate	Relative contribution of the group to overall CD rate
**1**	2297	356	24.20%	15.50%	3.75%	11.67%
**2**	1408	540	14.84%	38.35%	5.69%	17.70%
**2a**	1267	399	13.35%	31.49%	4.20%	13.08%
**2b**	141	141	1.49%	100%	1.49%	4.62%
**3**	2239	78	23.59%	3.48%	0.82%	2.56%
**4**	901	158	9.49%	17.54%	1.66%	5.18%
**4a**	801	58	8.44%	7.24%	0.61%	1.90%
**4b**	100	100	1.05%	100%	1.05%	3.28%
**5**	1097	822	11.56%	74.93%	8.66%	26.94%
**5.1**	862	589	9.08%	68.33%	6.21%	19.31%
**5.2**	235	233	2.48%	99.15%	2.46%	7.64%
**6**	300	300	3.16%	100%	3.16%	9.83%
**7**	163	160	1.72%	98.16%	1.69%	5.24%
**8**	293	232	3.09%	79.18%	2.44%	7.60%
**9**	36	36	0.38%	100%	0.38%	1.18%
**10**	756	369	7.97%	48.81%	3.89%	12.09%

Collected data of all births and caesarean deliveries performed at the department of obstetrics at Medical University Graz in the years 2017–2019 analysed using the ten group classification system by M. Robson.

**Fig 1:**
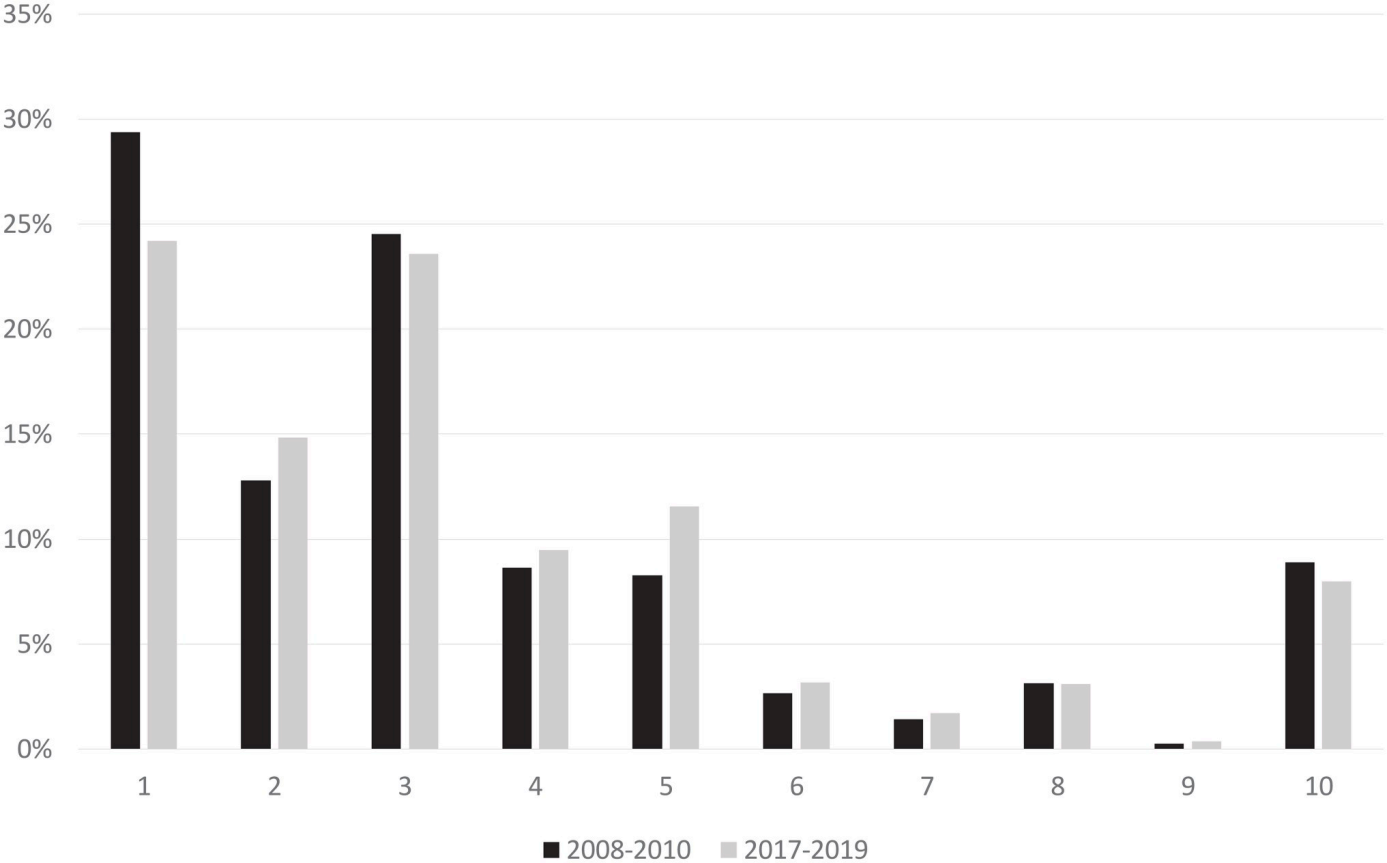
Group size. Comparison of the total number of births in each group between the years 2008–2010 and 2017–2019 using the ten group classification system by M. Robson (data in percentage).

The largest number of births per group was observed in groups 1,2 and 3, accounting for 66.7% in 2008–2010 and 62.6% in 2017–2019 (Fig 1).

Group 1 overall decreased in size attributing for 29.39% in 2008–2010 and only 24.2% in 2017–2019 (p<.001). Furthermore, more births were observed in Group 2 with 12.78% vs. 14.84% (p<.001). Both subgroups significantly increased from 11.68% to 13.35% (p<.05) in group 2a, as well as from 1.1% to 1.5% in 2b (p<.001).

A significant increase in birthrate in Group 5 was observed rising from 8.3% to 11.6% (p<.001).

The birthrate in group 10 decreased with 8.9% in 2008–2010 vs. 8% in 2017–2019 (p<.05) while the CD rate remained constant. (Fig 1).

The overall CD rate in 2008–2010 was 29.1% and in 2017–2019 32.2% (p<.05).

**Fig 2:**
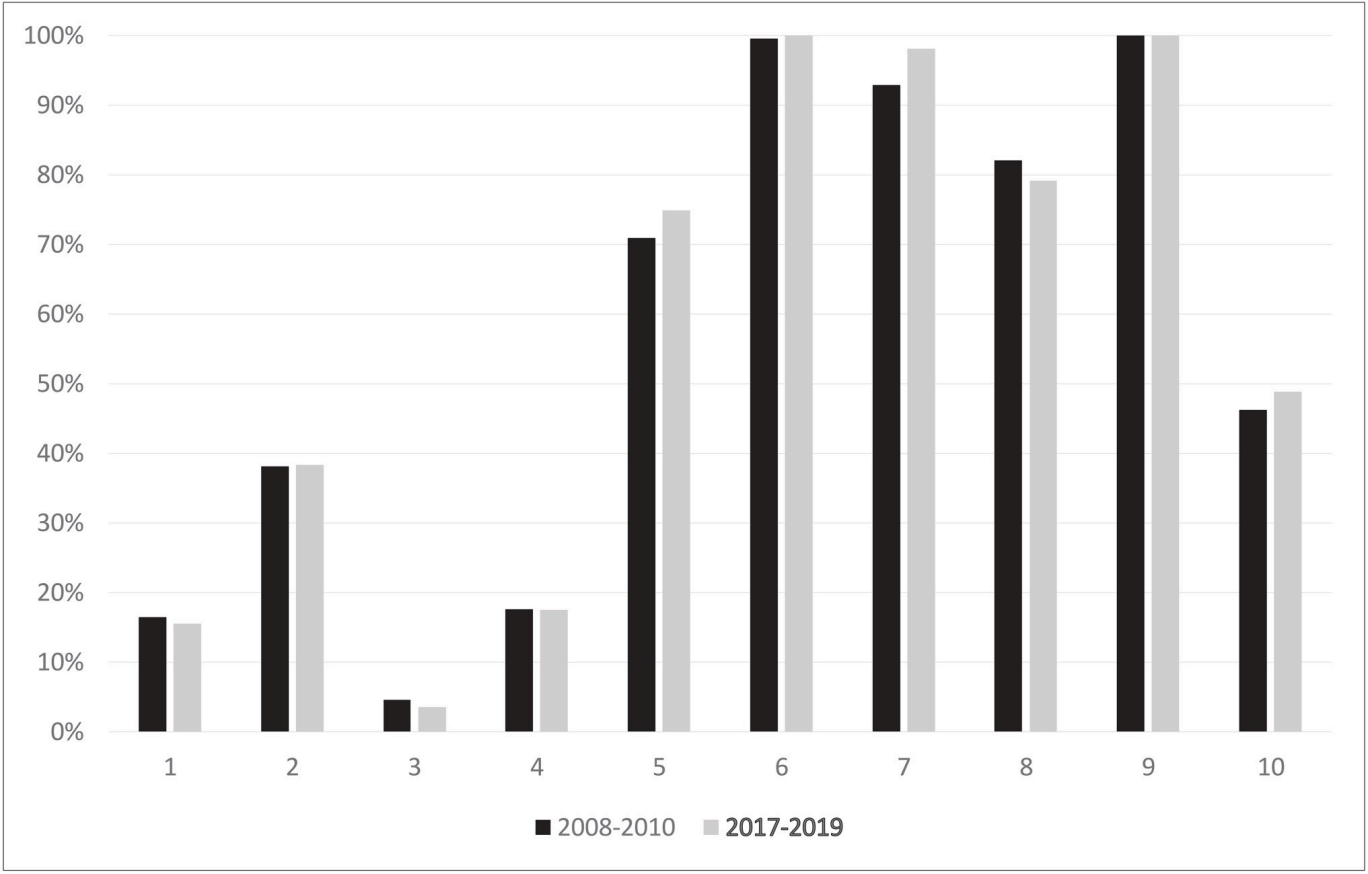
Caesarean delivery rate in each group. Comparison of the caesarean delivery rate in each group between the years 2008–2010 and 2017–2019 using the ten group classification system by M. Robson (data in percentage).

The largest contributors to the overall CD rate was in both observed periods Group 5 accounting for 20.2% and 26.9% of all procedures (p<.001). The CD rate in this group increased from 70.9% to 74.9% (Fig 2).

**Fig 3:**
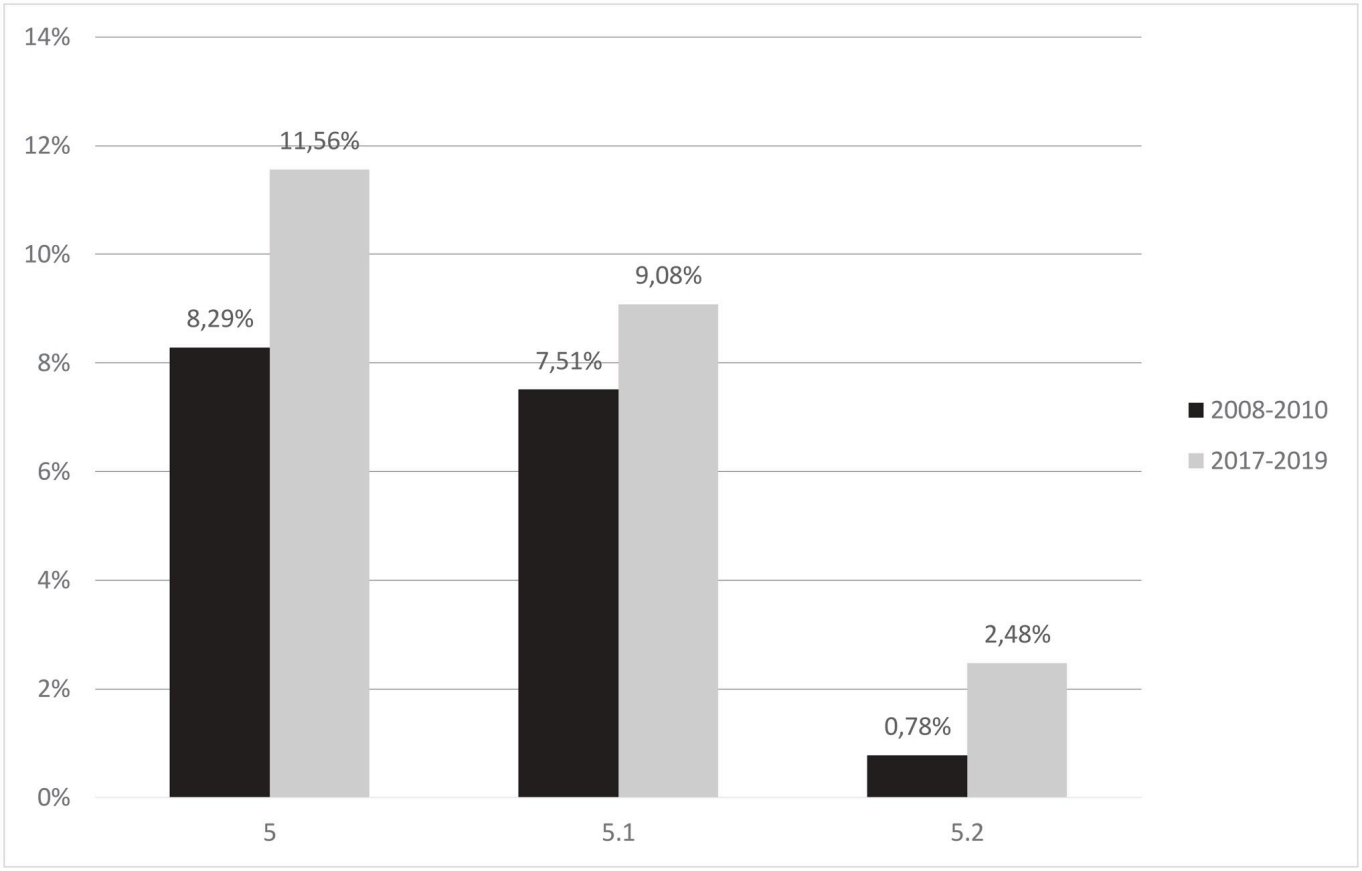
Group 5 and its subgroups 5.1 and 5.2. Comparison of size of Robson Group 5 and its subgroups between the years 2008–2010 and 2017–2019 (data in percentage).

Furthermore, the subgroup 5.1 (women with 1 previous CD) increased in size from 7.5% to 9.1% (p<.001), its CD rate remained constant 67.9% vs. 68.3% (Fig 3).

Group 2 was the second largest contributor to the overall CD rate with 16.8% vs. 17.7% in 2017–2019. Group 1 added 16.6% to the overall CD rate in 2008–2010 in contrast to 2017–2019 where it accounted for only 11.7% (p<.001).

**Fig 4:**
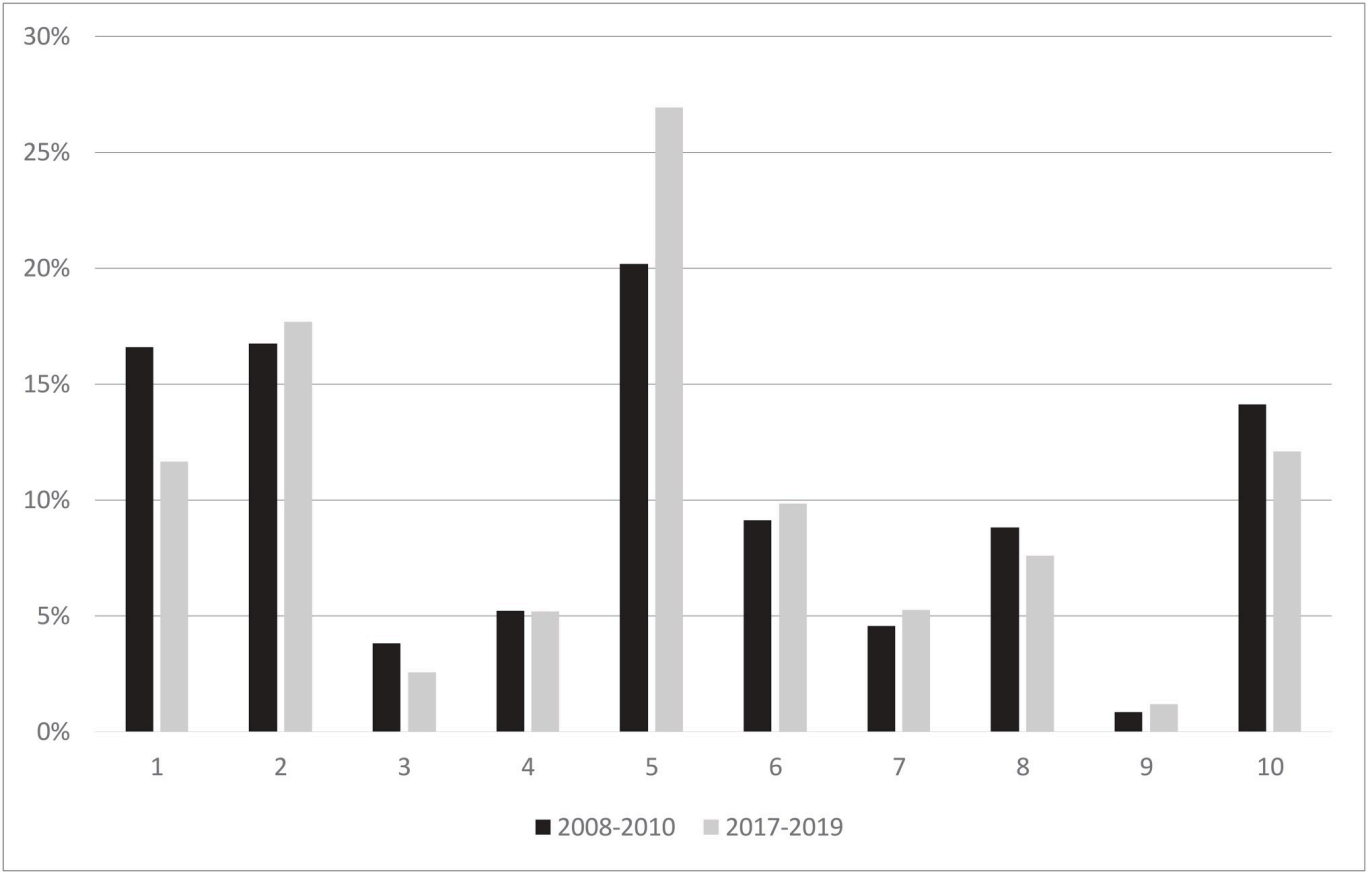
Relative contribution of each group. Comparison of the relative contribution of each group to overall caesarean delivery rate between the years 2008–2010 and 2017–2019 (data in percentage) calculated by dividing the number of caesarean deliveries in each group with the number of all caesarean deliveries in the observed time period.

Group 10 also added a large part to the overall rate with 14.1% and 12.1% in 2017–2019. Its group CD rate is slowly but continuously approaching 50%. (Fig 4).

Breech deliveries were only occasionally done in both time periods with decreasing numbers in the later period (9/321 vs. 3/463, p<.001). (Tables 2 and 3).

CD rates in women with multiple pregnancies (Group 8) decreased slightly from 82.04% vs. 79.18% without reaching significance (p = 0.784). (Fig 2).

## Discussion

The number of all births in the observed periods show an overall increase of deliveries in the municipality of Styria, Austria.

In the first period observed, the overall CD rate in our hospital was 29.1%. This rate is substantially higher than the worldwide rate with 18% and higher than the average European rate of 25% established in 2016 [2]. In the following years the CD rate increased up to 32.2%. This is consistent with rising CD rates in the whole country which reached a rate of 29.7% in 2017 [7].

The obstetric unit at MUG provides tertiary care for all high-risk pregnancies. The hospital is housing the biggest neonatal care unit in the region resulting in the management and delivery of most early preterm births (>23 weeks). The care for these difficult cases is often multidisciplinary and not seldom ends with an indication for delivery that does not allow time for labour induction. Therefore, a high CD rate is partially due to this patient population.

Our study showed that the three major contributors to the overall high CD rate in our hospital were group 5, 2 and 1 in 2008–2010 and group 5, 2 and 10 in 2017–2019.

When taking a look at group 10 (women with a single vertex pregnancy at < 37 weeks gestation, including women with a uterine scar) it can be observed that the overall number of births has decreased 0.9% in a 10 year period, the CD rate in this group however has remained constant with 46.2% and 48.8%. This might be due to better diagnostics and ultrasound skills which provide a better monitoring of the unborn foetuses and thus resulting in pregnancies being able to be prolonged.

The CD rate in group 10 at our hospital is approaching 50%. This is in contrast with findings of Brennan et al who analysed CD rates in 9 hospitals, 7 of them being tertiary centers and found a mean CD rate of 35.28% [8]. Furthermore Crosby D et al [9] also analysed CD rates in a tertiary hospital in Dublin and found a CD rate under 40%.

The mode of delivery in preterm infants is a widely discussed topic worldwide as well as in the German speaking countries. A retrospective study conducted at the Medical University of Vienna in 2017 demonstrated a 73.5% CD rate amongst singleton preterm deliveries from 23 + 0 to 33 + 6 gestational weeks. Despite its limitations, it showed a tendency toward better neonatal outcomes in small for gestational age infants (from 23+0 till 30+6 gestational weeks) as well as a benefit in appropriate for gestational week infants (from 23+0 till 27+6 gestational weeks) [10]. Furthermore in 2016 the guidelines for management of premature infants at the border of viability published by Austrian Society of Paediatrics and Adolescent Medicine clearly stated that in extreme preterm infants caesarean delivery is associated with better neonatal outcomes and a planned caesarean delivery should be the method of choice [11]. Although the Austrian Society for Gynaecology and Obstetrics (OEGGG) [12] did not agree with the before mentioned statement both articles illustrate a possible influence on the general mindset regarding preterm delivery mode in Austria.

The data regarding delivery mode in early preterm babies is unclear. Since the indications for preterm delivery are heterogeneous and often seem to not allow enough time for cervical ripening, clinicians not seldom turn to CD as mode for delivery. However, most studies that were conducted could not prove a benefit for neonatal outcome when comparing CD vs. vaginal delivery [13–15]. Furthermore all of this studies observed a significantly higher risk for maternal complications in CD [14]. Therefore international guidelines recommend that the decision regarding delivery mode should be made individually with the patient and depending on the clinical setting [12,16].

Indications for CD in group 10 should be discussed regularly especially when considering that a higher CD rate in this group contributes to a higher rate in group 5 in the future.

Group 2 comprises nulliparous women with a single vertex pregnancy, at ≥37 weeks gestation, who had labour induced or who had CD before labour. This group was the second largest contributor to the overall CD rate. The size of this group has increased significantly over 10 years while the CD rate in the group remained at 38%. Although both subgroups grew significantly larger the CD rate in group 2a remained constant.

This rate is consistent with previous reports [9,17,18]. It has been suggested by Robson that a ratio between group 1 and 2 lower than 2:1 suggests a higher incidence of induction and CD before labour [19]. In 2008–2010 the ratio was 2.3:1 however in 2017–2019 it dropped to 1.6:1. This suggests a high increase in interventions in nulliparous women which also influence the future size of group 5 and is similar to findings from Crosby D et al [9].

Up until 2018 induction of labour without medical indication was considered to be associated with higher risk for CD [20] however Grobman et al [21] showed there is no difference comparing to expectant management after 39 weeks of gestation.

When taken in account the patients from group 4a who were induced the overall rate of labour induction was 19.5% in 2008–2010 and 21.8% in 2017–2019 (p<.05). Although the number of induced patients rose the CD rate in both groups remained the same or rather showed a tendency to decrease in group 4a.

This trend is consistent with findings in other European countries [22].

Induction of labour is an intervention which should be utilized carefully and indications need to be analysed critically. Since at most departments the indications for primary caesarean delivery are being regularly discussed at team meetings, the indications for labour induction are seldomly addressed.

Group 5 was in both observed time periods the greatest contributor to the overall CD rate accounting for 20.18% (2008–2010) and 26.94% (2017–2019) of all CD.

These findings are consistent with CD rates reported from Canada [23], Ireland [9] and France [24] thus in both countries group 5 accounted for over 30% of all CD. The group on itself has grown from 8.3% to 11.6%. When taking a look at the subgroups 5.1 and 5.2 it becomes clear that both are responsible for the overall increase whilst the women with only 1 previous CD represent the majority of the births in group 5. This can be of course interpreted as a result of the continuously rising CD rates in the last decades. However, since also group 5.2 keeps increasing it should be discussed whether trial of labour after caesarean delivery (TOLAC) could be increased thus resulting in more vaginal births after caesarean delivery (VBAC) [25]. Strategies for motivating women towards TOLAC should be optimized.

Similar observations have been reported in a retrospective study from Ireland, which identified Groups 5, 2 and 1 as the largest contributors to the overall CD rate [9]. The main difference, however, remains the size of group 10, which was significantly lower in Ireland with 3.8% in 2014 in comparison to the reported 8% in 2017–2019 at our department. Furthermore, the CD rate in this group was in Ireland well below 40% at our hospital, however, keeps continuously approaching 50% (48.8% in 2017–2019). Further investigations are needed to identify reasons for these dissimilarities.

## Conclusion

The comparison of birth and caesarean rates at two timepoints after a 10-year period in our hospital shows a significantly higher overall CD rate which is mostly due to a rising number of births in group 5 (multiparous women, with at least one previous uterine scar with a single vertex pregnancy at ≥37 weeks gestation).

The indications for labour induction and primary CD which contribute to the size of group 2 (nulliparous women with a single vertex pregnancy, at ≥37 weeks gestation, who had labour induced or who had CD before labour) need to be examined critically since the size of this group significantly increased in the 10 year period thus contributing a large part to the overall CD rate.

Although the birthrate in group 10 (all women with a single vertex pregnancy at < 37 weeks gestation, including women with a uterine scar) decreased, the CD rate keeps approaching 50%.

Better strategies need to be developed to motivate women towards TOLAC after one previous caesarean delivery.

Regular discussions should be held regarding indications for labour induction and primary caesarean delivery in nulliparous women at term.

Frequent case analysis is necessary to determine whether 50% of all preterm births need to be delivered per caesarean delivery.

## References

[1] Todman D. A history of caesarean section: From ancient world to the modern era. Australian and New Zealand Journal of Obstetrics and Gynaecology. 2007. 10.1111/j.1479-828X.2007.00757.x 17877591

[2] Betrán AP, Ye J, Moller AB, Zhang J, Gülmezoglu AM, Torloni MR. The increasing trend in caesarean section rates: Global, regional and national estimates: 1990–2014. PLoS One. 2016 10.1371/journal.pone.0148343 26849801 PMC4743929

[3] Linton A, Peterson MR, Williams TV. Effects of maternal characteristics on cesarean delivery rates among U.S. Department of Defense Healthcare Beneficiaries, 1996–2002. Birth. 2004 10.1111/j.0730-7659.2004.0268.x 15015987

[4] Keag OE, Norman JE, Stock SJ. Long-term risks and benefits associated with cesarean delivery for mother, baby, and subsequent pregnancies: Systematic review and meta-analysis. PLoS Med. 2018 10.1371/journal.pmed.1002494 29360829 PMC5779640

[5] WHO Statement on caesarean section rates. Reproductive health matters. 2015. 10.1016/j.rhm.2015.07.007 26278843

[6] Robson MS. Can we reduce the caesarean section rate? Best Pract Res Clin Obstet Gynaecol. 2001 10.1053/beog.2000.0156 11359322

[7] Kirchengast S, Hartmann B. Recent lifestyle parameters are associated with increasing caesarean section rates among singleton term births in Austria. Int J Environ Res Public Health. 2019 10.3390/ijerph16010014 30577604 PMC6338883

[8] Brennan DJ, Robson MS, Murphy M, O’Herlihy C. Comparative analysis of international cesarean delivery rates using 10-group classification identifies significant variation in spontaneous labor. Am J Obstet Gynecol. 2009 10.1016/j.ajog.2009.06.021 19733283

[9] Crosby DA, Murphy MM, Segurado R, Byrne F, Mahony R, Robson M, et al Cesarean delivery rates using Robson classification system in Ireland: What can we learn? Eur J Obstet Gynecol Reprod Biol. 2019 10.1016/j.ejogrb.2019.03.011 30904815

[10] Holzer I, Lehner R, Ristl R, Husslein PW, Berger A, Farr A. Effect of delivery mode on neonatal outcome among preterm infants: an observational study. Wien Klin Wochenschr. 2017 10.1007/s00508-016-1150-2 28004267 PMC5599430

[11] Berger A, Kiechl-Kohlendorfer U, Berger J, Dilch A, Kletecka-Pulker M, Urlesberger B, et al Erstversorgung von Frühgeborenen an der Grenze der Lebensfähigkeit. Monatsschrift Kinderheilkd. 2017.

[12] Fischer T, Mörtl M, Reif P, Kiss H, Lang U. Statement by the OEGGG with Review of the Literature on the Mode of Delivery of Premature Infants at the Limit of Viability. Geburtshilfe Frauenheilkd. 2018 10.1055/a-0669-1480 30655647 PMC6294639

[13] Racusin DA, Antony KM, Haase J, Bondy M, Aagaard KM. Mode of Delivery in Premature Neonates: Does It Matter? AJP Rep. 2016 10.1055/s-0036-1585577 27468363 PMC4958016

[14] Ghi T, Maroni E, Arcangeli T, Alessandroni R, Stella M, Youssef A, et al Mode of delivery in the preterm gestation and maternal and neonatal outcome. J Matern Neonatal Med. 2010 10.3109/14767051003678259 20230325

[15] Werner EF, Han CS, Savitz DA, Goldshore M, Lipkind HS. Health outcomes for vaginal compared with cesarean delivery of appropriately grown preterm neonates. In: Obstetrics and Gynecology. 2013 10.1097/AOG.0b013e3182918a7e 23812452 PMC4700506

[16] NICE. Preterm labour and birth | Guidance and guidelines. NICE Guid Guidel. 2015.

[17] Vila-Candel R, Martín A, Escuriet R, Castro-Sánchez E, Soriano-Vidal FJ. Analysis of caesarean section rates using the robson classification system at a university hospital in Spain. Int J Environ Res Public Health. 2020 10.3390/ijerph17051575 32121364 PMC7084406

[18] Vogel JP, Betrán AP, Vindevoghel N, Souza JP, Torloni MR, Zhang J, et al Use of the robson classification to assess caesarean section trends in 21 countries: A secondary analysis of two WHO multicountry surveys. Lancet Glob Heal. 2015 10.1016/S2214-109X(15)70094-X 25866355

[19] Robson M. The ten group classification system (TGCS)-a common starting point for more detailed analysis. BJOG: An International Journal of Obstetrics and Gynaecology. 2015 10.1111/1471-0528.13267 25600521

[20] Smith DC, Phillippi JC, Lowe NK, Breman RB, Carlson NS, Neal JL, et al Using the Robson 10-Group Classification System to Compare Cesarean Birth Utilization Between US Centers With and Without Midwives. J Midwifery Women’s Heal. 2020 10.1111/jmwh.13035 31553129 PMC7024566

[21] Grobman WA, Rice MM, Reddy UM, Tita ATN, Silver RM, Mallett G, et al Labor induction versus expectant management in low-risk nulliparous women. N Engl J Med. 2018 10.1056/NEJMoa1800566 30089070 PMC6186292

[22] Einarsdóttir K, Sigurðardóttir H, Ingibjörg Bjarnadóttir R, Steingrímsdóttir Þ, Smárason AK. The Robson 10-group classification in Iceland: Obstetric interventions and outcomes. Birth. 2019 10.1111/birt.12415 30628120

[23] Roberge S, Dubé E, Blouin S, Chaillet N. Reporting Caesarean Delivery in Quebec Using the Robson Classification System. J Obstet Gynaecol Canada. 2017 10.1016/j.jogc.2016.10.01 28343556

[24] Lafitte AS, Dolley P, Le Coutour X, Benoist G, Prime L, Thibon P, et al Rate of caesarean sections according to the Robson classification: Analysis in a French perinatal network–Interest and limitations of the French medico-administrative data (PMSI). J Gynecol Obstet Hum Reprod. 2018 10.1016/j.jogoh.2017.11.012 29208502

[25] Royal College of Obstetricians and Gynaecologists. Birth after previous caesarean birth. R Coll Obstet Gynaecol. 2015.

